# Body composition parameters were associated with response to abiraterone acetate and prognosis in patients with metastatic castration‐resistant prostate cancer

**DOI:** 10.1002/cam4.5640

**Published:** 2023-02-07

**Authors:** Zhi‐Bin Ke, Qi You, Yu‐Ting Xue, Jiang‐Bo Sun, Jia‐Yin Chen, Wen‐Qi Liu, Yong Wei, Qing‐Shui Zheng, Xiao‐Dong Li, Xue‐Yi Xue, Ning Xu

**Affiliations:** ^1^ Department of Urology, Urology Research Institute, the First Affiliated Hospital Fujian Medical University Fuzhou China; ^2^ Department of Urology, National Regional Medical Center, Binhai Campus of the First Affiliated Fujian Medical University Fuzhou China; ^3^ Fujian Key Laboratory of Precision Medicine for Cancer, the First Affiliated Hospital Fujian Medical University Fuzhou China

**Keywords:** abiraterone acetate, body composition parameters, metastatic castration‐resistant prostate cancer, prognosis, therapeutic response

## Abstract

**Objective:**

To investigate the predictive value of body composition parameters for biochemical response to abiraterone acetate (AA) in metastatic castration‐resistant prostate cancer (mCRPC) patients with prior chemohormonal therapy.

**Methods:**

We retrospectively evaluated the clinicopathologic information of 132 mCRPC cases receiving AA treatment after chemohormonal therapy at hormone‐sensitive stage from July 2018 to June 2021. All patients were divided into AA responders and non‐responders according to the biochemical response to AA (prostate‐specific antigen (PSA) reduction ≥50% than pretreatment). Multivariate Logistic analysis was used to determine the independent predictors and develop predictive model of biochemical response to AA. Cox regression analysis was utilized to investigate the prognostic factors for time to biochemical progression (TTBP), radiological progression‐free survival (rPFS), failure‐free survival (FFS), and overall survival (OS) after AA treatment.

**Results:**

There were 57 AA responders and 75 AA non‐responders. Periprostatic fat area/prostate area (PPFA/PA) was decreased and skeletal muscle index (SMI) was increased in AA responders compared with AA non‐responders. Multivariable logistic analysis demonstrated that ADT duration ≥12 months, bone metastasis only, high SMI and low PPFA/PA were independent predictors of biochemical response to AA treatment. The FFS, TTBP, rPFS, and OS of patients with lower SMI or higher PPFA/PA was decreased compared with that of patients with higher SMI or lower PPFA/PA, respectively. Combining SMI, PPFA/PA, ADT duration and metastatic sites performed well in differentiating AA responders from non‐responders.

**Conclusions:**

High SMI and low PPFA/PA could predict biochemical response to AA treatment and preferable prognosis in mCRPC patients with prior chemohormonal therapy at hormone‐sensitive stage.

## Abstract 2

This study aimed to investigate the predictive value of body composition parameters for biochemical response to abiraterone acetate (AA) in metastatic castration‐resistant prostate cancer (mCRPC) patients with prior chemohormonal therapy. We found that high SMI and low PPFA/PA could predict biochemical response to AA treatment and preferable prognosis in mCRPC patients with prior chemohormonal therapy at hormone‐sensitive stage.

## 1 INTRODUCTION

As the most frequently diagnosed tumor in aging men, prostate cancer (PCa) has become the second leading cause of cancer death, with the proportion of diagnosed distant‐stage PCa from 3.9% to 8.2% in 2007 to 2018.^1^, ^2^ It has been widely known that androgen deprivation therapy (ADT) is the main treatment throughout holistic management of PCa owing to its dependence on androgen for progression.^3^ Depending on tumor sensibility to ADT, metastatic PCa is divided into two stages, metastatic hormone‐sensitive prostate cancer (mHSPC) and metastatic castration‐resistant prostate cancer (mCRPC).^3^ The therapeutic effect of chemohormonal therapy (chemotherapy in combination with ADT) at the hormone‐sensitive stage has been established significantly improving overall survival (OS) in the study of STAMPEDE and CHAARTED.^4^, ^5^ However, after a period of chemohormonal therapy, most mHSPC patients would be inevitably transitioned into the castration‐resistant state, which indicated a significantly increased possibility of death from PCa and markedly worse prognosis.^6^

Abiraterone acetate (AA), an androgen inhibitor of the cytochrome P450 c17 enzyme complex, was confirmed to prolong survival of mCRPC patients after chemotherapy in COU‐AA‐301, and currently is one of the most commonly used treatment options in mCRPC patients.^7^ Most mCRPC patients initially respond to AA treatment; nevertheless, approximately one third of patients show primary resistance without any decrease in prostate‐specific antigen (PSA) levels.^8^ Moreover, secondary resistance would eventually happen to almost all patients who initially exhibit a response to AA.^7^, ^8^, ^9^ Although patients with mCPRC now have many approved treatment options, the optimal sequencing pathway is currently unknown, which is aggravated by primary and secondary resistance.^10^ Hence, it is urgently needed to seek predictors of therapeutic response to AA in mCRPC patients. Recently, several markers were shown to be associated with therapeutic response to AA in mCRPC patients, for example, androgen receptor splice variant‐7, chromogranin A, and neuron‐specific enolase, etc.^8^, ^11^ However, most of them were unpractical and not fully validated, and there is still a lack of convenient and effective clinical predictors of the therapeutic response to AA treatment in mCRPC patients.

Recently, the role of body composition on the prediction of therapeutic efficacy and cancer prognosis has been increasingly recognized.^12^, ^13^ For example, our previous study revealed that preoperative lower relative visceral fat area was a vital independent predictor of response to intravesical Bacillus Calmette‐Guerin immunotherapy and was associated with preferable prognosis in patients with non‐muscle invasive bladder cancer.^12^ Sarcopenia, characterized by progressive and generalized loss of skeletal muscle mass and strength, was showed to be associated with PSA progression in mHSPC patients receiving early docetaxel or AA treatment.^14^ A high volume of subcutaneous adipose tissue was correlated with a preferable prognosis in mCRPC patients treated with AA.^15^ Low skeletal muscle volume was confirmed as an independent adverse prognostic factor for the progression of disease in patients with mCRPC treated with docetaxel.^16^ However, most study focused on the impact of body composition on survival outcome of mCRPC, and the relationship between body composition profiles and biochemical response to AA in the special metastatic populations who developed castration resistance after chemohormonal therapy at hormone sensitive stage remains unaddressed.

In current study, we aimed to investigate whether body composition parameters prior to AA treatment could accurately predict biochemical response to AA treatment and survival outcome in mCRPC patients after chemohormonal therapy at hormone‐sensitive stage.

## 2 MATERIALS AND METHODS

### 2.1 Patients and data collection

This study was approved by the Ethics Committee of the First Affiliated Hospital of Fujian Medical University and written informed consents were provided by all cases. The clinicopathologic data of 132 mCRPC cases receiving AA treatment from July 2018 to June 2021 in the First Affiliated Hospital of Fujian Medical University were retrospectively collected. According to the Prostate Cancer Clinical Trials Working Group guidelines, CRPC was diagnosed as follows^17^: after initial continuous ADT treatment reaching the castration level (serum testosterone <50 ng/dl or <1.7 nmol/L), serum PSA increased (>2 ng/ml or by more than 50%, for three consecutive times at an interval of 1 week) or new lesions appeared (≥2 new bone lesions or a soft tissue lesion).

Inclusion criteria were as follows: (1) pathological diagnosis of prostate cancer by biopsy and existence of high‐volume tumor according to CHAARTED study^5^; (2) presence of bone or visceral metastatic lesions by imageological examination; (3) progression to mCRPC after chemohormonal therapy (ADT plus docetaxel‐based chemotherapy) at the hormone sensitive stage^18^; (4) complete clinicopathologic data. Patients who met all of the above criteria were included in the study.

Exclusion criteria were as follows: (1) less than six cycles of docetaxel‐based chemotherapy; (2) history of radiation, immunotherapy or targeted therapy; (3) irregular AA treatment; (4) combined with other malignant tumors; (5) history of hypophysis or adrenal cortex dysfunction; (6) adverse effects of level 3–4 causing the cessation of chemotherapy or AA treatment; (7) history of chronic or acute liver disease or abnormal transaminase levels; (8) abnormal bone marrow function or renal function; (9) The Eastern Cooperative Oncology Group (ECOG) performance status score > 2; (10) incomplete clinicopathologic data. Patients who met any of the above criteria were excluded from the study.

The clinicopathologic features were collected for analysis, including age, body mass index (BMI), Eastern Cooperative Oncology Group performance status score (ECOG score), International Society of Urological Pathology (ISUP) grading group, clinical T stage, prior ADT duration before AA treatment, PSA as diagnosis, PSA at AA start, PSA nadir after AA treatment, metastatic sites, body composition parameters (including skeletal muscle index (SMI), visceral fat area (VFA), subcutaneous fat area,^19^ total fat area (TFA), relative visceral fat area (rVFA), periprostatic fat area (PPFA), periprostatic fat area/prostate area (PPFA/PA), periprostatic fat thickness (PPFT), periprostatic fat thickness/subcutaneous fat thickness (PPFT/SFT), biochemical progression, and radiological progression).

### 2.2 Body composition parameters

Computerized tomography (CT), positron emission tomography/computed tomography (PET/CT), and magnetic resonance imaging (MRI) images of mCRPC patients were all acquired before AA treatment. We employed Image J v1.51 k software (https://imagej.nih.gov/ij/, Wayne Rasband, USA) to quantify abdominal muscle and adipose characteristics at the third lumbar vertebra (L3) using CT or PET/CT images because this anatomical location is strongly associated with whole‐body volume. We preset the thresholds of Hounsfield units (HU) as −190 to −30 HU for fat tissue and −29 and 150 HU for skeletal muscle as previously reported.^12^, ^20^ Prostate area (PA) and PPFA, PPFT and subcutaneous fat thickness (SFT) were also delineated and calculated from MRI using Image J v1.51 k software.^21^, ^22^ Two researchers (Zhi‐Bin Ke and Qi You) received training on how to accurately obtain segment muscle and adipose tissues, and inconsistencies were addressed by discussion.

### 2.3 Assessment of treatment response

The primary endpoint of this study was biochemical response to AA treatment. A reduction of at least 50% in comparison with pretreatment was defined as a biochemical response to AA, as previously described.^18^, ^23^ Other secondary endpoint included time to biochemical progression (TTBP), radiological progression‐free survival (rPFS), failure‐free survival (FFS), and OS after AA treatment. Biochemical progression was defined as an increase of PSA by 25% and excluded from PSA flare phenomenon compared with pretreatment.^24^ PSA flare phenomenon was defined as any initial increase and followed by a decrease of PSA level during AA treatment.^25^ The rPFS was defined as time to ≥2 new lesions on an 8‐week bone scan plus two additional lesions on a confirmatory scan, ≥2 new confirmed lesions on any scan ≥12 weeks after AA treatment, and/or progression in nodes or viscera on cross‐sectional imaging, or death.^26^ The FFS was defined as time to evidence of at least one of the following: biochemical progression or radiological progression or death from PCa.^27^ The OS was defined as time to death from any cause. Duration of AA treatment was determined from the time from first AA dose to treatment discontinuation owing to any reason including death from any causes, disease progression, or intolerable adverse events.^28^

### 2.4 Protocols of abiraterone acetate treatment and follow‐up

AA was administered orally at 1000 mg once a day, and prednisone was administered orally at 5 mg twice a day. Once adverse effects of level 3–4 occurred (for example, hypertension, hypokalemia, edema, or other non‐corticosteroid toxic events, etc.), AA treatment was immediately suspended until the toxic symptoms were relieved to level 1 or lower. Liver/renal function, electrolytes, blood routine examination, and blood pressure were measured regularly every 1–3 months during AA treatment to timely detect adverse events caused by AA treatment.

In our center, biochemical follow‐up was routinely performed every 2–3 months. Radiological follow‐up was performed by whole‐body radionuclide bone imaging, or CT/MRI examination, or ^18^F‐FDG PET/CT, or ^68^Ga‐PSMA‐11 PET/CT. Most patients conducted traditional imaging follow‐up (for example, whole‐body radionuclide bone imaging or CT/MRI examination) based on the condition or patient needs. Considering that the exorbitant price of PET/CT, only those presenting emerging severe symptoms or biochemical progression or radiological progression indicated by traditional imaging would be recommended for ^18^F‐FDG PET/CT (Before the ^68^Ga‐PSMA‐11 PET/CT is available in our hospital) or ^68^Ga‐PSMA‐11 PET/CT. Patients were followed up by outpatient and telephone. The median follow‐up time was 8.9 months (range 3.5–17.2 months).

### 2.5 Statistical method

Statistical analysis was performed using SPSS 26.0 software (IBM Corp., Armonk, NY, USA) or R ×64 4.1.0 software. All normally distributed data were expressed as the mean ± SD^29^ and analyzed using Student's *t*‐test while non‐normally distributed data were expressed as median (minimum–maximum) and analyzed using the Kruskal–Wallis test. The Wilcoxon test was used to compared the paired continuous data. Numerical data were analyzed using the chi‐square test or Fisher's test. Spearman method was used to conduct correlation analysis. Multivariate Logistic regression analysis was used to determine the independent predictors, and develop predictive model of biochemical response to AA treatment in mCRPC patients. Univariate and multivariate Cox analyses were utilized to investigate the independent prognostic factors for FFS, TTBP, rPFS, and OS after AA treatment in mCRPC patients. *p* Value <0.05 in univariate analysis was considered as a threshold for selecting variables into multivariate analysis. The Kaplan–Meier method was utilized for survival analysis and log‐rank test was utilized for comparison. The GraphPad Prim 7.0 was used to draw survival curve. The receiver operating characteristic (ROC) curve was utilized to identify the optimal cut‐off value according to maximal Youden index. DeLong’ test was used to compare the area under ROC curve (AUC). MedCalc version 20 or R ×64 4.1.0 software was used to draw ROC curve and calculate AUC. *p* Value <0.05 was considered statistically significant.

## 3 RESULTS

### 3.1 Patient characteristics

In this study, we enrolled a total of 132 mCRPC patients with complete clinicopathologic data into final analysis, including 57 AA responders and 75 AA non‐responders. The baseline clinicopathologic characteristics of all patients were showed in Table 1. The median age was 71 years old and the median BMI was 23.88 kg/m^2^. The median PSA level at abiraterone start was 10.37 ng/ml. There was no significant difference in age, BMI, ECOG score, ISUP grading group, clinical T‐stage, PSA as diagnosis, and PSA at AA start between these two groups (*p* > 0.05). ADT duration ≥12 months and bone metastasis only were more common in AA responders compared with non‐responders (*p* < 0.05). There was a total of 98 patients experiencing biochemical progression (responders vs. non‐responders, 54.39% vs. 89.33%) and 87 patients experiencing radiological progression (responders vs. non‐responders, 43.86% vs. 82.67%) (Table 2).

**TABLE 1:** Baseline clinicopathologic characteristics before AA treatment

Variables	Whole cohort (*N* = 132)
Age (years)	71 (36–85)
BMI (kg/m^2^)	23.88 (18.51–30.86)
ECOG score	
0 or 1	86 (65.15%)
2	46 (34.85%)
ISUP grading group	
1	12 (9.09%)
2	22 (16.68%)
3	34 (25.75%)
4	45 (34.09%)
5	19 (14.39%)
Clinical T stage	
2	21 (15.91%)
3	55 (41.67%)
4	56 (42.42%)
ADT duration before AA	
<12 months	67 (50.76%)
≥12 months	65 (49.24%)
Metastatic sites	
Bone only	64 (48.48%)
Viscera	68 (51.52%)
PSA at diagnosis (ng/ml)	13.10 (4.10–96.00)
PSA at abiraterone start (ng/ml)	10.37 (1.11–91.76)
SMI (cm^2^/m^2^)	49.71 (32.15–76.55)
VFA (cm^2^)	126.65 (55.85–216.53)
SFA (cm^2^)	134.52 (76.55–201.32)
TFA (cm^2^)	255.83 (150.21–385.54)
rVFA	0.48 (0.33–0.58)
PPFA (cm^2^)	12.65 (5.52–21.47)
PPFA/PA	0.94 (0.42–2.50)
PPFT (mm)	9.25 (1.23–16.59)
PPFT/SFT	0.32 (0.05–0.72)

Abbreviations: AA, abiraterone acetate; ADT, androgen deprivation therapy; BMI, body mass index; ECOG, Eastern Cooperative Oncology Group performance status score; SFA, subcutaneous fat area; ISUP, International Society of Urological Pathology; PPFA/PA, periprostatic fat area/prostate area; PPFA, periprostatic fat area; PPFT/SFT, periprostatic fat thickness/subcutaneous fat thickness; PPFT, periprostatic fat thickness; PSA, prostate‐specific antigen; rVFA, relative visceral fat area; SMI, skeletal muscle index; TFA, total fat area; VFA, visceral fat area.

**TABLE 2:** Comparison of clinicopathologic characteristics between AA responders and non‐responders

Variables	AA responders (*N* = 57)	AA non‐responders (*N* = 75)	*p* Value
Age (years)	71.00 (51.00–85.00)	71.50 (36.00–84.00)	0.716
BMI (kg/m^2^)	23.72 (13.51–30.86)	24.16 (18.72–29.74)	0.385
ECOG score			
0 or 1	38 (66.67%)	48 (64%)	0.750
2	19 (33.33%)	27 (36%)	
ISUP grading group			
1	7 (12.28%)	5 (6.67%)	0.206
2	12 (21.05%)	10 (13.33%)	
3	17 (29.82%)	17 (22.67%)	
4	14 (24.57%)	31 (41.33%)	
5	7 (12.28%)	12 (16%)	
Clinical T stage			
2	7 (12.28%)	14 (18.67%)	0.284
3	28 (49.12%)	27 (36%)	
4	22 (38.60%)	34 (45.33%)	
ADT duration before AA			
<12 months	16 (28.07%)	51 (68%)	<0.001
≥12 months	41 (71.93%)	24 (32%)	
Metastatic sites			
Bone only	39 (68.42%)	25 (33.33%)	<0.001
Viscera	18 (31.58%)	50 (66.67%)	
PSA at diagnosis (ng/ml)	12.93 (4.78–42.40)	13.00 (4.10–96.00)	0.448
PSA at abiraterone start (ng/ml)	9.53 (1.11–91.76)	9.33 (1.64–77.07)	0.889
PSA nadir after AA treatment (ng/ml)	1.27 (0.02–10.81)	8.865 (1.04–48.12)	<0.001
PSA reduction (%)	84.77 (55.08–99.51)	20.80 (−87.10–46.94)	<0.001
SMI (cm^2^/m^2^)	51.84 (40.18–76.55)	45.96 (32.15–61.17)	<0.001
VFA (cm^2^)	112.65 (55.85–216.53)	134.53 (79.67–194.64)	0.005
SFA (cm^2^)	134.52 (82.35–185.43)	134.56 (76.55–201.32)	0.772
TFA (cm^2^)	250.85 (150.21–384.09)	271.33 (165.50–385.54)	0.107
rVFA	0.46 (0.33–0.57)	0.49 (0.38–0.57)	0.001
PPFA (cm^2^)	11.16 (5.52–18.29)	14.56 (7.95–21.47)	<0.001
PPFA/PA	0.80 (0.42–1.56)	1.39 (0.66–2.50)	<0.001
PPFT (mm)	8.32 (1.25–16.32)	9.58 (1.23–16.59)	0.013
PPFT/SFT	0.30 (0.08–0.53)	0.34 (0.05–0.72)	0.01
Biochemical progression			
Yes	31 (54.39%)	67 (89.33%)	<0.001
No	26 (45.61%)	8 (10.67%)	
Radiological progression			
Yes	25 (43.86%)	62 (82.67%)	<0.001
No	32 (56.14%)	13 (17.33%)	

Abbreviations: AA, abiraterone acetate; ADT, androgen deprivation therapy; BMI, body mass index; ECOG, Eastern Cooperative Oncology Group performance status score; ISUP, International Society of Urological Pathology; PPFA/PA, periprostatic fat area/prostate area; PPFA, periprostatic fat area; PPFT/SFT, periprostatic fat thickness/subcutaneous fat thickness; PPFT, periprostatic fat thickness; PSA, prostate‐specific antigen; rVFA, relative visceral fat area; SFA, subcutaneous fat area; SMI, skeletal muscle index; TFA, total fat area; VFA, visceral fat area.

There were moderate correlations between VFA, SFA, TFA, PPFA, and BMI (Spearman's correlation coefficients ≥0.4 and <0.7). Specially, there was a strong correlation between abdominal visceral adipose area and periprostatic adipose area (Spearman's correlation coefficients ≥0.7). In contrast, skeletal muscle parameter presented weak correlations with BMI as well as adipose tissues (Spearman's correlation coefficients <0.4). (Table S1). Moreover, there were weak correlations of body composition parameters and serum nutritional markers, including albumin, hemoglobin, and prognostic nutritional index (Spearman's correlation coefficients <0.4).

### 3.2 Predictive value of body composition parameters for biochemical response to AA treatment

We compared the body composition parameters between AA responders and non‐responders. The results showed that VFA, rVFA, PPFA, PPFA/PA, PPFT, and PPFT/SFT was significantly lower in AA responders compared with non‐responders (*p* < 0.05), and that SMI was significantly higher in AA responders compared with non‐responders (*p* < 0.05). However, the SFA and TFA were not significantly different between AA responders and non‐responders (*p* > 0.05). (Table 2).

**FIGURE 1:**
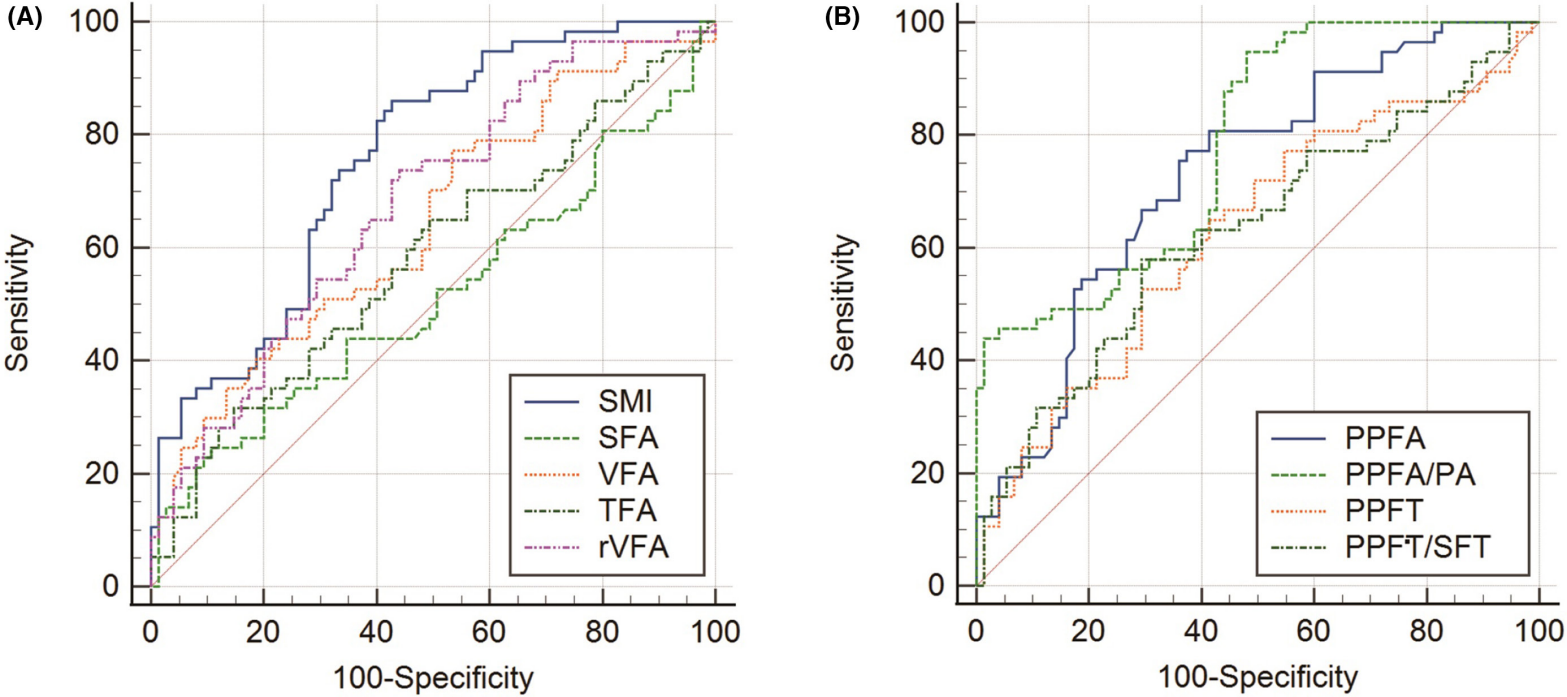
ROC curve to determine the optimal cutoff value and the predictive ability of abdominal muscle and adipose characteristics (A), and periprostatic fat parameters (B).

The AUC for biochemical response to AA was 0.756 for SMI, 0.643 for VFA, 0.515 for SFA, 0.582 for TFA, 0.676 for rVFA, 0.729 for PPFA, 0.784 for PPFA/PA, 0.627 for PPFT, 0.630 for PPFT/SFT, indicating that SMI and PPFA/PA were superior to other body composition parameters in predicting the biochemical response to AA treatment. The optimal cut‐off value for SMI and PPFA/PA was 46.471 and 1.301, respectively (Figure 1 and Table S2). Hence, we divided all patients into high and low SMI group, high and low PPFA/PA group in subsequent analysis.

Next, considering multicollinearity, preceding results (*p* < 0.05) and previous reports, we merely included SMI, PPFA/PA, ADT duration before AA treatment and metastatic sites into further multivariate Logistic analysis. Multivariable Logistic analysis demonstrated that ADT duration ≥12 months (*p* = 0.016, OR = 3.232, 95%CI 1.249–8.363), bone metastasis only (*p* = 0.006, OR = 0.269, 95%CI 0.106–0.683), high SMI (*p* = 0.013, OR = 3.785, 95%CI 1.318–10.866) and low PPFA/PA (*p* < 0.001, OR = 0.054, 95%CI 0.013–0.215) were all independent predictors of biochemical response to AA treatment in mCRPC patients with prior chemohormonal therapy at hormone‐sensitive stage. The results showed that SMI and PPFA/PA prior treatment is of great importance in predicting AA response in mCRPC patients with prior chemohormonal therapy at hormone‐sensitive stage. (Table 3).

**TABLE 3:** Multivariate Logistic regression analysis for predictors associated with biochemical response to AA treatment in mCRPC patients after chemohormonal therapy at hormone‐sensitive stage

Variables	OR	95%CI	*p* Value
ADT duration (<12 vs. ≥ 12 months)	3.232	1.249–8.363	0.016
Metastatic sites (bone only vs. viscera)	0.269	0.106–0.683	0.006
SMI group (low vs. high)	3.785	1.318–10.866	0.013
PPFA/PA group (low vs. high)	0.054	0.013–0.215	<0.001

Abbreviations: AA, abiraterone acetate; ADT, androgen deprivation therapy; CI, confidence interval; mCRPC, metastatic castration‐resistant prostate cancer; OR, odds ratio; PPFA/PA, periprostatic fat area/prostate area; SMI, skeletal muscle index.

We compared the clinicopathologic characteristics between high and low SMI group. The results revealed that low SMI was associated with higher ISUP grading group, higher clinical T‐stage, higher proportion of ADT duration <12 months and visceral metastasis, higher PSA reduction, higher proportion of biochemical, and radiological progression. (*p* < 0.05, Table 4) We also compared the clinicopathologic characteristics between high and low PPFA/PA group. The results revealed that high PPFA/PA was associated with higher BMI, worse performance status, higher clinical T‐stage, higher proportion of ADT duration <12 months and visceral metastasis, higher proportion of biochemical, and radiological progression. (*p* < 0.05, Table 5).

**TABLE 4:** Comparison of clinicopathologic characteristics between high and low SMI

Variables	Low SMI (*N* = 51)	High SMI (*N* = 81)	*p* Value
Age (years)	73 (36–81)	69 (51–85)	0.474
BMI (kg/m^2^)	23.60 (18.72–29.30)	24.21 (18.51–30.86)	0.103
ECOG score			
0 or 1	33 (64.71%)	53 (65.43%)	0.932
2	18 (35.29%)	28 (34.57%)	
ISUP grading group			
1	0 (0%)	12 (14.81%)	<0.001
2	3 (5.88%)	19 (23.46%)	
3	8 (15.69%)	26 (32.10%)	
4	26 (50.98%)	19 (23.46%)	
5	14 (27.45%)	5 (6.17%)	
Clinical T stage			
2	6 (11.76%)	15 (18.52%)	0.010
3	15 (29.41%)	40 (49.38%)	
4	30 (58.82%)	26 (32.10%)	
ADT duration before AA			
<12 months	41 (80.39%)	26 (32.10%)	<0.001
≥12 months	10 (19.61%)	55 (67.90%)	
Metastatic sites			
Bone only	15 (29.41%)	49 (60.49%)	0.001
Viscera	36 (70.59%)	32 (39.51%)	
PSA at diagnosis (ng/ml)	13.10 (4.10–96.00)	13.30 (4.78–57.10)	0.944
PSA at abiraterone start (ng/ml)	11.23 (1.11–91.76)	9.48 (1.12–77.07)	0.383
PSA nadir after AA treatment (ng/ml)	3.37 (0.02–39.28)	7.88 (0.11–48.12)	0.019
PSA reduction (%)	29.05 (−64.83–98.34)	2.61 (−87.10–99.51)	<0.001
Biochemical progression			
Yes	47 (92.16%)	51 (62.96%)	<0.001
No	4 (7.84%)	30 (37.04%)	
Radiological progression			
Yes	43 (84.31%)	42 (51.85%)	<0.001
No	8 (15.69%)	39 (48.15%)	

Abbreviations: AA, abiraterone acetate; ADT, androgen deprivation therapy; BMI, body mass index; ECOG, Eastern Cooperative Oncology Group performance status score; ISUP, International Society of Urological Pathology; PSA, prostate‐specific antigen; SMI: skeletal muscle index.

**TABLE 5:** Comparison of clinicopathologic characteristics between high and low PPFA/PA

Variables	Low PPFA/PA (*N* = 90)	High PPFA/PA (*N* = 42)	*p* Value
Age (years)	71 (51–84)	68 (36–85)	0.833
BMI (kg/m^2^)	23.58 (18.51–30.64)	24.96 (20.76–29.30)	0.012
ECOG score			
0 or 1	64 (71.11%)	22 (52.38%)	0.035
2	26 (28.89%)	20 (47.62%)	
ISUP grading group			
1	9 (10%)	3 (7.14%)	0.311
2	19 (21.11%)	3 (7.14%)	
3	27 (30%)	7 (16.67%)	
4	26 (28.89%)	19 (45.24%)	
5	9 (10%)	10 (23.81%)	
Clinical T stage			
2	15 (16.67%)	6 (14.9%)	0.001
3	46 (51.11%)	9 (21.43%)	
4	29 (32.22%)	27 (64.28%)	
ADT duration before AA			
<12 months	38 (42.22%)	29 (69.05%)	0.004
≥12 months	52 (57.78%)	13 (30.95%)	
Metastatic sites			
Bone only	49 (54.44%)	15 (35.71%)	0.045
Viscera	41 (45.56%)	27 (64.29%)	
PSA at diagnosis (ng/ml)	13.10 (4.59–56.64)	13.05 (4.10–96.00)	0.792
PSA at abiraterone start (ng/ml)	9.76 (1.11–91.76)	12.60 (2.34–77.07)	0.267
PSA nadir after AA treatment (ng/ml)	2.96 (0.02–40.00)	8.97 (1.25–48.12)	<0.001
PSA reduction (%)	67.47 (−64.83–99.51)	28.22 (−87.10–90.20)	<0.001
Biochemical progression			
Yes	61 (67.78%)	37 (88.10%)	0.013
No	29 (32.22%)	5 (11.90%)	
Radiological progression			
Yes	47 (52.22%)	35 (83.33%)	0.001
No	43 (47.78%)	7 (16.67%)	

Abbreviations: AA, abiraterone acetate; ADT, androgen deprivation therapy; BMI, body mass index; ECOG, Eastern Cooperative Oncology Group performance status score; ISUP, International Society of Urological Pathology; PPFA/PA, periprostatic fat area/prostate area; PSA, prostate‐specific antigen.

### 3.3 Prognostic value of body composition parameters in mCRPC patients after AA treatment

For OS, univariable Cox regression analysis revealed that lower age, lower PSA nadir after AA treatment, ADT duration ≥12 months, high SMI and low PPFA/PA were associated with longer OS (*p* < 0.05); multivariate Cox regression analysis demonstrated that lower age (*p* = 0.004, HR = 1.065, 95%CI 1.021–1.110), lower PSA nadir after AA treatment (*p* = 0.004, HR = 1.045, 95%CI 1.014–1.077), high SMI (*p* < 0.001, HR = 0.250, 95%CI 0.119–0.524), and low PPFA/PA (*p* < 0.001, HR = 3.204, 95%CI 1.696–6.053) were the independent prognostic factors of longer OS after AA treatment in mCRPC patients with prior chemohormonal therapy at hormone‐sensitive stage. (Table 6).

**TABLE 6:** Univariate and multivariate Cox regression analyses exploring prognostic factors for OS in mCRPC patients receiving AA treatment

Variables	OS			
	Univariate		Multivariate	
	HR (95% CI)	*p* Value	HR (95% CI)	*p* Value
Age (years)	1.044 (1.004–1.086)	0.031	1.065 (1.021–1.110)	0.004
BMI (kg/m^2^)	1.012 (0.903–1.135)	0.834	–	–
ECOG score (0/1 vs. 2)	0.875 (0.455–1.679)	0.687	–	–
ISUP grading group				
1	Ref		–	–
2	1.779 (0.368–8.600)	0.474		
3	2.389 (0.535–10.668)	0.254		
4	3.277 (0.754–14.235)	0.113		
5	1.971 (0.359–10.806)	0.435		
Clinical T stage				
2	Ref		–	–
3	0.483 (0.225–1.036)	0.061		
4	0.549 (0.251–1.200)	0.133		
PSA at AA start (ng/ml)	0.998 (0.979–1.018)	0.879	–	–
PSA nadir after AA (ng/ml)	1.055 (1.026–1.084)	<0.001	1.045 (1.014–1.077)	0.004
ADT duration (<12 vs. ≥12 months)	0.315 (0.164–0.605)	0.001	–	0.148
Metastatic sites (bone only vs. viscera)	1.637 (0.892–3.006)	0.112	–	0.623
SMI (low vs. high)	0.200 (0.098–0.406)	<0.001	0.250 (0.119–0.524)	<0.001
PPFA/PA (low vs. high)	4.005 (2.151–7.459)	<0.001	3.204 (1.696–6.053)	<0.001

Abbreviations: AA, abiraterone acetate; ADT, androgen deprivation therapy; BMI, body mass index; CI, confidence interval; ECOG, Eastern Cooperative Oncology Group performance status score; HR, hazard ratio; ISUP, International Society of Urological Pathology; mCRPC, metastatic castration‐resistant prostate cancer; OS, overall survival; PPFA/PA, periprostatic fat area/prostate area; PSA, prostate‐specific antigen; SMI, skeletal muscle index.

For TTBP, univariable Cox regression analysis revealed that lower PSA nadir after AA treatment, ADT duration ≥12 months, bone metastasis only, high SMI and low PPFA/PA were associated with longer TTBP (*p* < 0.05); multivariate Cox regression analysis demonstrated that ADT duration ≥12 months (*p* = 0.001, HR = 0.449, 95%CI 0.282–0.714), high SMI (*p* < 0.001, HR = 0.297, 95%CI 0.180–0.490) and low PPFA/PA (*p* = 0.010, HR = 1.818, 95%CI 1.153–2.866) were the independent prognostic factors of longer TTBP after AA treatment in mCRPC patients. (Table S3).

For rPFS and FFS, univariable Cox regression analysis revealed that lower ISUP grading group, lower PSA nadir after AA treatment, ADT duration ≥12 months, bone metastasis only, high SMI and low PPFA/PA were associated with longer rPFS (*p* < 0.05); multivariate Cox regression analysis demonstrated that lower PSA nadir after AA treatment (rPFS: *p =* 0.035, HR = 1.023, 95%CI 1.002–1.046; FFS: *p =* 0.037, HR = 1.023, 95%CI 1.001–1.045), ADT duration ≥12 months (rPFS: *p =* 0.001, HR = 0.392, 95%CI 0.221–0.695; FFS *p =* 0.003, HR = 0.503, 95%CI 0.317–0.797), high SMI (rPFS: *p* < 0.001, HR = 0.256, 95%CI 0.143–0.459; FFS: *p* < 0.001, HR = 0.404, 95%CI 0.252–0.648) and low PPFA/PA (rPFS: *p =* 0.039, HR = 1.663, 95%CI 1.025–2.697; FFS: *p =* 0.031, HR = 1.616, 95%CI 1.043–2.502) were independent prognostic factors of longer rPFS and FFS after AA treatment in mCRPC patients. (Tables S4 and S5).

**FIGURE 2:**
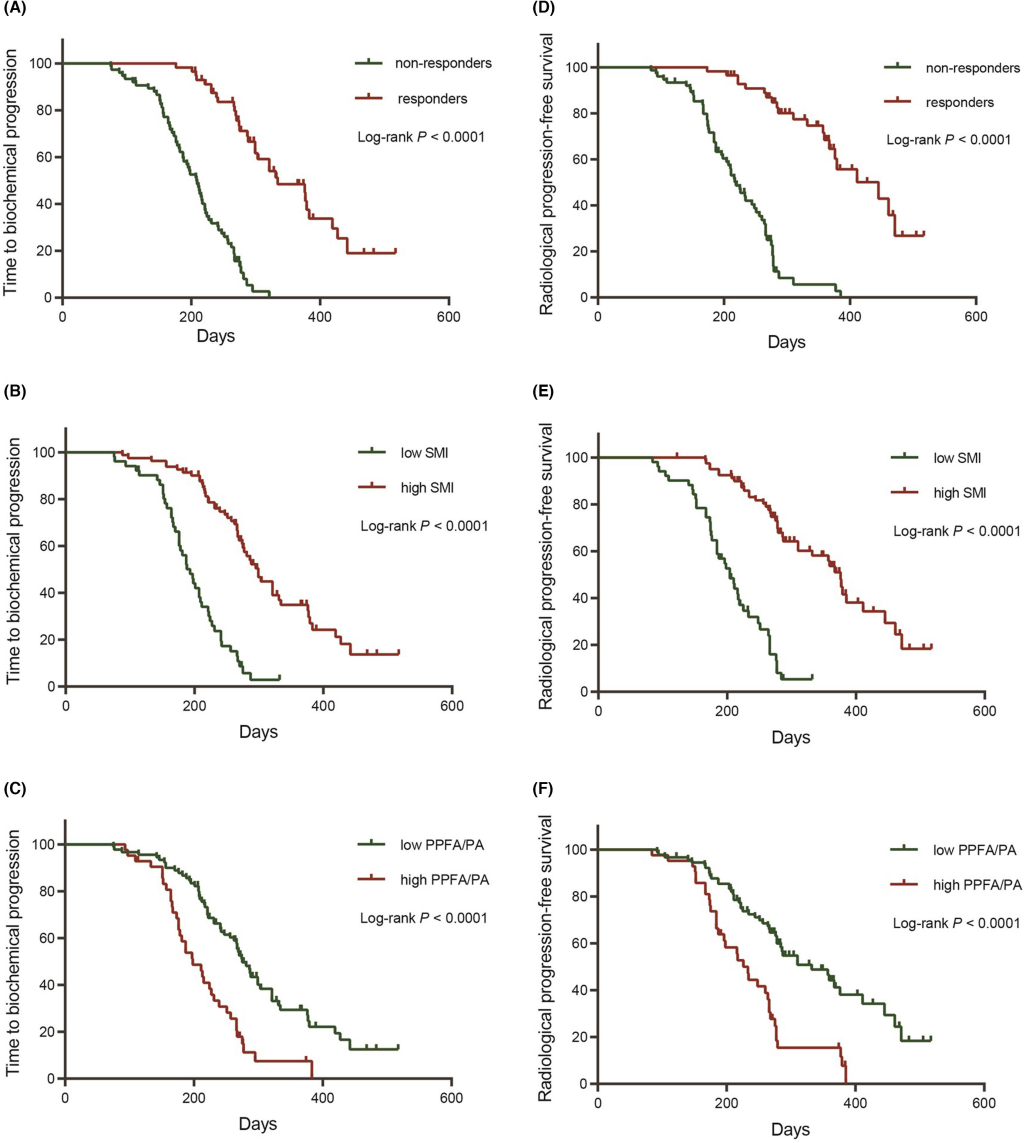
The Kaplan–Meier survival analysis to demonstrate the difference in time to biochemical progression (TTBP) and radiological progression free survival (rPFS) between AA responders and non‐responders (A, D), high and low SMI group (B, E), high and low PPFA/PA group (C, F), respectively.

**FIGURE 3:**
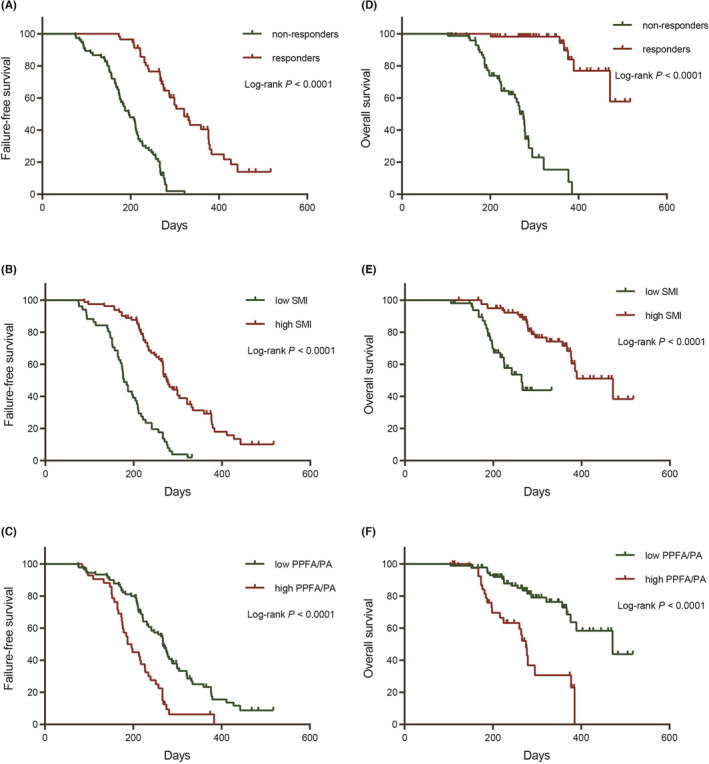
The Kaplan–Meier survival analysis to demonstrate the difference in failure‐free survival (FFS), and overall survival (OS) between AA responders and non‐responders (A, D), high and low SMI group (B, E), high and low PPFA/PA group (C, F), respectively.

The Kaplan–Meier survival analysis suggested that the FFS, TTBP, rPFS, and OS of AA responders were significantly higher compared with that of AA non‐responders. We divided all cases into high and low SMI group, high and low PPFA/PA group, according to the optimal cut‐off value. Survival analysis demonstrated that the FFS, TTBP, rPFS, and OS of patients with lower SMI or higher PPFA/PA were significantly decreased compared with these of patients with higher SMI or lower PPFA/PA, respectively (*p* < 0.05, Figures 2 and 3).

### 3.4 A body composition model for predicting biochemical response to AA treatment

We divided all patients into two groups according to the time of stating AA treatment: training group (July 2018–June 2020) and testing group (July 2020–June 2021). Then, we utilized multivariate Logistic regression method to develop predictive models for biochemical response to AA treatment in mCRPC patients with prior chemohormonal therapy at hormone‐sensitive stage. Model A was composed of SMI group and PPFA/PA group; model B was composed of SMI group, PPFA/PA group and ADT duration before AA treatment; model C was composed of SMI group, PPFA/PA group, ADT duration before AA treatment and metastatic sites.

**FIGURE 4:**
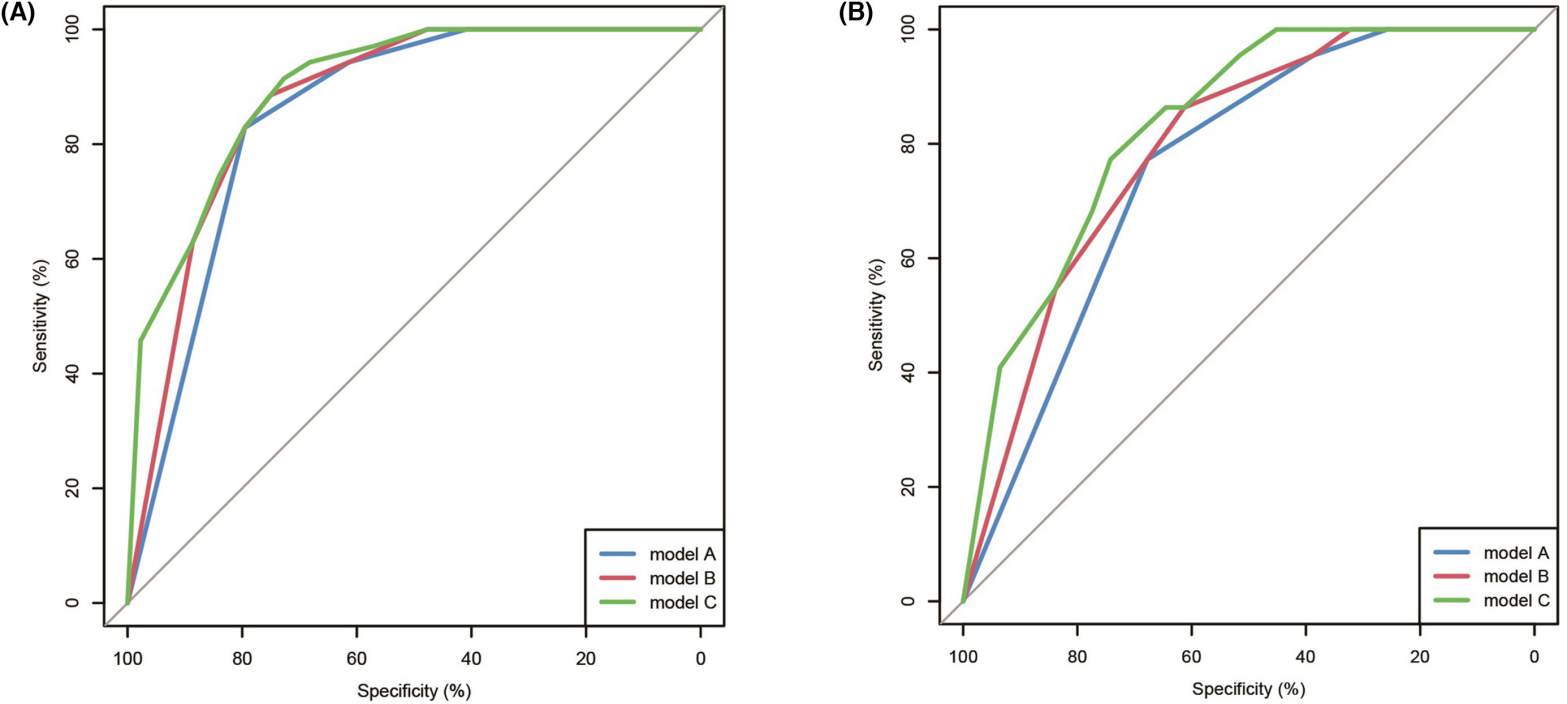
ROC curve to reveal the predictive ability of three model for predicting biochemical response to abiraterone acetate treatment in training (A) and testing cohort (B).

The AUC of model A, model B, and model C was 0.854, 0.875, 0.899 in training group, and 0.760, 0.794, 0.832 in testing group, respectively. DeLong’ test revealed that the difference of AUC between model A and B was not statistically significant (*p* > 0.05). However, the AUC of model C was significantly increased compared with model A in both training and testing group (*p* = 0.018 and 0.037, respectively). Therefore, combining SMI group, PPFA/PA group, ADT duration before AA treatment and metastatic sites performed well in distinguishing AA responders from non‐responders in patients with mCRPC after chemohormonal therapy at hormone‐sensitive stage. (Figure 4).

## 4 DISCUSSION

In the past decade, the application of novel androgen receptor signaling inhibitors had completely changed the treatment landscape of mCRPC, especially abiraterone and enzalutamide.^30^ The COU‐AA‐301 trial had demonstrated that AA treatment could significantly improve the overall outcome of mCRPC patients after treating with docetaxel.^31^ However, primary and secondary resistance remains a troublesome problem although AA exhibited the efficacy in some mCRPC male with prior chemohormonal therapy.^7^ How to early predict the biochemical response to AA before starting AA treatment in patients with mCRPC after chemohormonal therapy at hormone‐sensitive stage is of great importance for urologist. Treatment using AA in non‐responders would delay optimal treatment opportunity and result in tumor progression, and early prediction of therapeutic response may enable early modification of therapeutic strategy.^32^ Previous studies have revealed that body composition would change during hormonotherapy. Pezaro et al. found that maximal androgen suppression was associated with loss of muscle and visceral fat.^33^ Fischer et al. uncovered that skeletal muscle area of mCRPC patients exhibited a significant reduction after abiraterone or enzalutamide treatment.^34^ Hanson et al. demonstrated that percent lean of mCRPC was lower than HSPC.^35^ However, up to date, there were no studies focusing on exploring whether body composition profiles before AA treatment could predict biochemical response to AA and prognosis in patients with mCRPC with prior chemohormonal therapy at hormone‐sensitive stage.

As far as we know, this is the first study investigating the relationship between body composition profiles and biochemical response to AA in the special metastatic populations who developed castration resistance after chemohormonal therapy at hormone sensitive stage. The results demonstrated that AA responders had higher SMI, and lower VFA, rVFA, PPFAPPFA/PA, PPFT, PPFT/SFT before starting AA treatment in comparison with AA non‐responders. The higher SMI and lower PPFA/PA before starting AA treatment were the vital independent predictors of biochemical response to AA treatment and were associated with increased FFS, TTBP, rPFS, and OS in mCRPC patients with prior chemohormonal therapy at hormone‐sensitive stage. Finally, we developed and validated a predictive model, which combined SMI group, PPFA/PA group, ADT duration before AA treatment and metastatic sites. This model performed well in distinguishing AA responders from non‐responders in the special metastatic populations developing castration resistance after chemohormonal therapy at hormone sensitive stage.

Recently, with the improvement of daily living conditions, the incidence of obesity has been increased continuously, which is considered as the most common metabolic diseases and to be related to the tumorigenesis of various cancers.^36^ Adipose tissue could increase the risk of tumor occurrence through secreting a variety of adipokines and inflammatory factors.^37^, ^38^ It has been demonstrated that patients with BMI >25 kg/m^2^ suffered a worse outcome with the treatment of AA in mCRPC,^39^, ^40^ which suggested that obesity might affect the therapeutic effect of AA. It was also reported that the obesity was associated with a higher risk for aggressive PCa and prostate cancer‐specific mortality.^41^ The uptake and storage of lipids, and formation of lipid droplets in PCa cells were vital to the occurrence and progression of PCa.^42^ Although BMI was the most frequently used indicator to evaluate obesity degree in previous studies, BMI was not able to accurately reflect the difference in fat and muscle distribution.^43^, ^44^ In our study, we revealed that BMI before AA treatment was similar between AA responders and non‐responders, indicating that BMI might not be an ideal indicator for predicting biochemical response to AA. Recently, using the latest imaging techniques, we could precisely delineate the distribution of various body anatomical composition, for example, visceral adipose tissue, subcutaneous adipose tissue, skeletal muscle area, etc.^38^ Previous studies have confirmed that the role of body composition profiles on the prediction of therapeutic efficacy of cancer patients.^12^ In our study, we first explored the role of body composition parameters before AA treatment in predicting biochemical response to AA in the special metastatic populations who developed castration resistance after chemohormonal therapy at hormone sensitive stage, and revealed for that first time that SMI, and PPFA/PA before starting AA treatment were associated with biochemical response to AA treatment.

Visceral fat tissue could secrete multiple adipokines and inflammatory cytokines, which might increase the risk and drive the development of malignant tumor.^45^ Periprostatic adipose tissue, which showed intimate anatomical relationship with visceral adipose, was regarded as the vital part of the prostate microenvironment and could influence the aggressiveness and development of PCa.^46^ Our study also found that there was a strong correlation between abdominal visceral fat area and periprostatic fat area, and that high PPFA/PA was associated with higher clinical T‐stage and higher proportion of biochemical/radiological progression, which was consistent with previous reports. It has been reported that the measurement of periprostatic fat, particularly PPFA/PA, could benefit to the accurate risk stratification of PCa to avoid dispensable biopsy.^47^ The PPFA/PA was also regarded as an independent predictor for lymph node metastasis in PCa patients undergoing radical prostatectomy.^48^ In men with mCRPC treating with docetaxel, high volume of visceral fat was related to the decrease of survival.^49^ Besides, the ratio of periprostatic fat thickness to subcutaneous fat thickness (PPFT/SFT) on MRI before treatment was an independent indicator of predicting the survival of males with hormone‐naïve advanced PCa.^47^ However, to our knowledge, there was no study exploring whether periprostatic fat was associated with the therapeutic response to AA treatment in patients with mCRPC. In this study, we revealed that VFA, rVFA, PPFA, PPFA/PA, PPFT, and PPFT/SFT was significantly lower in AA responders compared with AA non‐responders. More importantly, our study innovatively found that periprostatic fat area/prostate area (PPFA/PA) was superior to other fat parameters (VFA, SFA, TFA, rVFA, PPFA, PPFT, and PPFT/SFT) in predicting biochemical response to AA treatment in mCRPC patients. Besides, low PPFA/PA could not only independently predict biochemical response to AA treatment, but also predict increased FFS, TTBP, rPFS and OS in the special metastatic populations who developed castration resistance after chemohormonal therapy at hormone sensitive stage.

Skeletal muscle is the protein reservoir and plays an irreplaceable role in regulating the glucose and lipid homeostasis.^50^ The significant progressive loss of skeletal muscle, which is called sarcopenia, has been considered as a nonnegligible disorder and is associated with increased adverse outcomes.^51^ However, there is still no universal definition of sarcopenia. SMI is the most widely used indicator to define sarcopenia in numerous studies^52^, ^53^ and has been demonstrated to be associated with the prognosis of various cancers.^54^ Gadducci et al^55^ revealed that the change of the baseline SMI during treatment was usually related to the progression‐free survival and OS of patients with gynecological cancer. Jang et al^56^ indicated that the change of skeletal muscle mass during treatment was considered as a reliable predictor of the chemotherapy toxicity as well as the OS rates. Taki et al.^57^ revealed that in elderly patients with gastric cancer, the preoperative SMI could predict the OS after gastrectomy. Among PCa patients after robot‐assisted radical prostatectomy, reduced skeletal muscle size was an indicator of postoperative urinary incontinence.^58^ Furthermore, Chiang et al^44^ indicated that each 1% decrease of SMI would lead to a 9% increase in risk of non‐cancer mortality in patients with high‐risk PCa. In patients with mHSPC who treated with early docetaxel or abiraterone acetate, sarcopenia was an independent prognostic predictor of the poor FFS.^59^ However, whether SMI before treatment was associated with the biochemical response of AA treatment in the special metastatic populations who developed castration resistance after chemohormonal therapy at hormone sensitive stage remained an unsolved issue. Our study first demonstrated that high SMI was an independent predictor of biochemical response to AA treatment and an independent prognostic factor of FFS, TTBP, rPFS and OS in patients with mCRPC.

Most importantly, we developed a novel predictive model, combining SMI group, PPFA/PA group, ADT duration before AA treatment, and metastatic sites, for biochemical response to AA treatment in mCRPC patients. The AUC of this predictive model was 0.899 in training group and 0.832 in testing group, respectively, which indicating that this predictive model performed well in distinguishing AA responders from non‐responders in patients with mCRPC after chemohormonal therapy at hormone‐sensitive stage. It has been reported that the prior duration of ADT was of great importance in the therapeutic response to subsequent therapy in mCRPC patients.^60^ In this study, we also found that prior ADT duration ≥12 months was an independent predictor of biochemical response to AA treatment and was associated with better FFS, TTBP, rPFS, and OS, which was consistent with previous study. Besides, previous studies also demonstrated that visceral metastasis was associated with the survival outcome of mCRPC patients receiving AA treatment.^61^ In our study, we also found that the extent of metastatic spread was an independent predictor of biochemical response to AA treatment in mCRPC patients; nevertheless, the extent of metastatic spread might not be related to the prognosis of mCRPC patients after treating with AA.

However, there are several limitations of this study. First of all, this is retrospective study with a limited sample size. Further multi‐center prospective study is required to demonstrate the associations of SMI and PPFA/PA with the therapeutic response to AA treatment. Second, due to the exorbitant price of PET/CT, only those presenting emerging severe symptoms or biochemical progression or radiological progression indicated by traditional imaging would be recommended for PET/CT examination. Hence, there were only approximately half of the patients possessing post‐treatment data of body composition profiles, and we could not conduct more accurate assessment of rPFS by comparing results of PET/CT before and after AA treatment. Finally, further researches into pathological and molecular biological mechanism of periprostatic adipose tissue and abdominal muscle distribution influencing the biochemical response of AA in mCRPC patients were required in future.

## 5 CONCLUSIONS

High SMI and low PPFA/PA were vital independent predictors of biochemical response to AA treatment and were associated with preferable prognosis in patients with mCRPC. Combining SMI, PPFA/PA, ADT duration before AA treatment and metastatic sites performed well in differentiating AA responders from non‐responders in patients with mCRPC after chemohormonal therapy at hormone‐sensitive stage.

## AUTHOR CONTRIBUTIONS

**Zhi‐Bin Ke:** Conceptualization (lead); writing – original draft (lead). **Qi You:** Investigation (equal); writing – original draft (equal). **Yu‐Ting Xue:** Methodology (equal); writing – original draft (equal). **Jiang‐Bo Sun:** Investigation (lead). **Jia‐Yin Chen:** Visualization (lead). **Wen‐Qi Liu:** Methodology (lead). **Yong Wei:** Data curation (lead). **Qing‐Shui Zheng:** Supervision (lead). **Xiao‐Dong Li:** Formal analysis (lead). **Xue‐Yi Xue:** Project administration (equal); supervision (equal); writing – review and editing (equal). **Ning Xu:** Project administration (lead); writing – review and editing (lead).

## FUNDING INFORMATION

The study was supported by the “Eyas Plan” Youth Top‐notch Talent Project of Fujian Province (Grant number: SCYJHBJRC‐XN2021), Class B Talent Research Project of the First Affiliated Hospital of Fujian Medical University (Grant number: YJCRC‐B‐XN2022), and Startup Fund for Scientific Research of Fujian Medical University (Grant number: 2021QH2034).

## CONFLICT OF INTEREST

All authors declare no conflict of interests.

## ETHICAL APPROVAL

This study was approved by the Ethics Committee of the First Affiliated Hospital of Fujian Medical University and all patients provided written informed consent.

## CONSENT FOR PUBLICATION

All authors read the final manuscript and agreed to publish it.

## Supporting information


Table S1

Table S2

Table S3

Table S4

Table S5

## Data Availability

The data presented in the study are included in the article/Supporting information, further inquiries can be directed to the corresponding authors.
