# Supplementary material for: Body composition parameters were associated with response to abiraterone acetate and prognosis in patients with metastatic castration‐resistant prostate cancer
Source: Cancer Med. 2023 Feb 7;12(7):8251–66. doi: 10.1002/cam4.5640 (PMC10134370; doi:10.1002/cam4.5640)
Supplement: Supplementary file 1 — Table S1 [file CAM4-12-8251-s003.docx]

| Table S1. Correlation analysis of body composition parameters | | | | | | | | | | |  |
| --- | --- | --- | --- | --- | --- | --- | --- | --- | --- | --- | --- |
| Correlation | BMI | SMI | VFA | SFA | TFA | rVFA | PPFA | PPFA/PA | PPFT | PPFT/SFT |  |
| BMI |  | 0.164 | 0.546 | 0.494 | 0.579 | 0.266 | 0.439 | 0.290 | 0.383 | 0.165 |  |
|  |  |  |  |  |  |  |  |  |  |  |  |
| SMI | 0.164 |  | -0.137 | -0.034 | -0.096 | -0.188 | -0.189 | -0.293 | -0.113 | -0.06 |  |
|  |  |  |  |  |  |  |  |  |  |  |  |
| VFA | 0.546 | -0.137 |  | 0.608 | 0.938 | 0.716 | 0.733 | 0.578 | 0.675 | 0.344 |  |
|  |  |  |  |  |  |  |  |  |  |  |  |
| SFA | 0.494 | -0.034 | 0.608 |  | 0.830 | -0.033 | 0.407 | 0.241 | 0.436 | 0.077 |  |
|  |  |  |  |  |  |  |  |  |  |  |  |
| TFA | 0.579 | -0.096 | 0.938 | 0.830 |  | 0.481 | 0.667 | 0.490 | 0.643 | 0.268 |  |
|  |  |  |  |  |  |  |  |  |  |  |  |
| rVFA | 0.266 | -0.188 | 0.716 | -0.033 | 0.481 |  | 0.550 | 0.472 | 0.461 | 0.419 |  |
|  |  |  |  |  |  |  |  |  |  |  |  |
| PPFA | 0.439 | -0.189 | 0.733 | 0.407 | 0.667 | 0.550 |  | 0.834 | 0.706 | 0.532 |  |
|  |  |  |  |  |  |  |  |  |  |  |  |
| PPFA/PA | 0.290 | -0.293 | 0.578 | 0.241 | 0.490 | 0.472 | 0.834 |  | 0.594 | 0.453 |  |
|  |  |  |  |  |  |  |  |  |  |  |  |
| PPFT | 0.383 | -0.113 | 0.675 | 0.436 | 0.643 | 0.461 | 0.706 | 0.594 |  | 0.773 |  |
|  |  |  |  |  |  |  |  |  |  |  |  |
| PPFT/SFT | 0.165 | -0.06 | 0.344 | 0.077 | 0.268 | 0.419 | 0.532 | 0.453 | 0.773 |  |  |
|  |  |  |  |  |  |  |  |  |  |  |  |

BMI: body mass index; SMI: skeletal muscle index; VFA: visceral fat area; SFA: subcutaneous fat area; TFA: total fat area; rVFA: relative visceral fat area; PPFA: periprostatic fat area; PPFA/PA: periprostatic fat area / prostate area; PPFT: periprostatic fat thickness; PPFT/SFT: periprostatic fat thickness / subcutaneous fat thickness.
