# Supplementary material for: Body composition parameters were associated with response to abiraterone acetate and prognosis in patients with metastatic castration‐resistant prostate cancer
Source: Cancer Med. 2023 Feb 7;12(7):8251–66. doi: 10.1002/cam4.5640 (PMC10134370; doi:10.1002/cam4.5640)
Supplement: Supplementary file 2 — Table S2 [file CAM4-12-8251-s004.docx]

| Table S2. ROC curve analyses of abdominal and prostatic fat for predicting biochemical response to AA treatment in mCRPC patients with prior chemohormonal therapy at hormone-sensitive stage | | | | | | | |
| --- | --- | --- | --- | --- | --- | --- | --- |
| Variables | AUC | 95%*CI* | Youden | Cut-off | Sensitivity | Specificity | *P* value |
| SMI (cm^2^/m^2^) | 0.756 | 0.674-0.827 | 0.4330 | 46.471 | 85.96 | 57.33 | <0.0001 |
| VFA (cm^2^) | 0.643 | 0.555-0.725 | 0.2386 | 135.232 | 77.19 | 46.67 | 0.0034 |
| SFA (cm^2^) | 0.515 | 0.426-0.603 | 0.1389 | 154.325 | 24.56 | 89.33 | 0.7806 |
| TFA (cm^2^) | 0.582 | 0.493-0.667 | 0.1691 | 221.159 | 31.58 | 85.33 | 0.1084 |
| rVFA | 0.676 | 0.589-0.755 | 0.2968 | 0.489 | 73.68 | 56.00 | 0.0002 |
| PPFA (cm^2^) | 0.729 | 0.645-0.803 | 0.3986 | 13.267 | 77.19 | 62.67 | <0.0001 |
| PPFA/PA | 0.784 | 0.704-0.851 | 0.4674 | 1.301 | 94.74 | 52.00 | <0.0001 |
| PPFT (mm) | 0.627 | 0.539-0.710 | 0.2358 | 9.274 | 64.91 | 58.67 | 0.0110 |
| PPFT/SFT | 0.630 | 0.542-0.713 | 0.2856 | 0.308 | 57.89 | 70.67 | 0.0091 |

ROC: receiver operating characteristic curve; AA: abiraterone acetate; AUC: area under the ROC curve; CI: confidence interval; SMI: skeletal muscle index; VFA: visceral fat area; SFA: subcutaneous fat area; TFA: total fat area; rVFA: relative visceral fat area; PPFA: periprostatic fat area; PPFA/PA: periprostatic fat area/prostate area; PPFT: periprostatic fat thickness; PPFT/SFT: periprostatic fat thickness/subcutaneous fat thickness.
