# Supplementary material for: Body composition parameters were associated with response to abiraterone acetate and prognosis in patients with metastatic castration‐resistant prostate cancer
Source: Cancer Med. 2023 Feb 7;12(7):8251–66. doi: 10.1002/cam4.5640 (PMC10134370; doi:10.1002/cam4.5640)
Supplement: Supplementary file 3 — Table S3 [file CAM4-12-8251-s005.docx]

| Table S3. Univariate and multivariate Cox regression analyses exploring prognostic factors for TTBP in mCRPC patients receiving AA treatment | | | | | |
| --- | --- | --- | --- | --- | --- |
| Variables | TTBP | | | | |
|  | Univariate | |  | multivariate | |
|  | *HR* (95% *CI*) | *P* value |  | *HR* (95% *CI*) | *P* value |
| Age (years) | 1.003(0.980-1.026) | 0.823 |  | - | - |
| BMI (kg/m^2^) | 0.969(0.900-1.043) | 0.398 |  | - | - |
| ECOG score (0/1 vs. 2) | 0.844(0.549-1.297) | 0.439 |  | - | - |
| ISUP grading group |  |  |  |  |  |
| 1 | Ref |  |  | - | - |
| 2 | 1.094(0.471-2.545) | 0.834 |  |  |  |
| 3 | 0.889(0.387-2.042) | 0.782 |  |  |  |
| 4 | 1.684(0.780-3.634) | 0.184 |  |  |  |
| 5 | 2.317(0.993-5.407) | 0.052 |  |  |  |
| Clinical T stage |  |  |  |  |  |
| 2 | Ref |  |  | - | - |
| 3 | 0.614(0.342-1.101) | 0.102 |  |  |  |
| 4 | 1.155(0.662-2.013) | 0.612 |  |  |  |
| PSA at AA start (ng/ml) | 1.000(0.987-1.013) | 0.970 |  | - | - |
| PSA nadir after AA (ng/ml) | 1.043(1.021-1.065) | ＜0.001 |  | - | 0.111 |
| ADT duration  (<12 vs. ≥12 months) | 0.321(0.211-0.489) | ＜0.001 |  | 0.449(0.282-0.714) | 0.001 |
| Metastatic sites  (bone only vs. viscera) | 1.609(1.077-2.403) | 0.020 |  | - | 0.966 |
| SMI group (low vs. high) | 0.222(0.142-0.349) | ＜0.001 |  | 0.297(0.180-0.490) | <0.001 |
| PPFA/PA (low vs. high) | 2.371(1.538-3.653) | ＜0.001 |  | 1.818(1.153-2.866) | 0.010 |

TTBP: time to biochemical progression; mCRPC: metastatic castration-resistant prostate cancer; AA: abiraterone acetate; HR: hazard ratio; CI: confidence interval; BMI: body mass index; ECOG: Eastern Cooperative Oncology Group performance status score; ISUP: International Society of Urological Pathology; PSA: prostate-specific antigen; ADT: androgen deprivation therapy; SMI: skeletal muscle index; PPFA/PA: periprostatic fat area/prostate area.
