# Supplementary material for: Body composition parameters were associated with response to abiraterone acetate and prognosis in patients with metastatic castration‐resistant prostate cancer
Source: Cancer Med. 2023 Feb 7;12(7):8251–66. doi: 10.1002/cam4.5640 (PMC10134370; doi:10.1002/cam4.5640)
Supplement: Supplementary file 4 — Table S4 [file CAM4-12-8251-s001.docx]

| Table S4. Univariate and multivariate Cox regression analyses exploring prognostic factors for rPFS in mCRPC patients receiving AA treatment | | | | | |
| --- | --- | --- | --- | --- | --- |
| Variables | rPFS | | | | |
|  | Univariate | |  | multivariate | |
|  | *HR* (95% *CI*) | *P* value |  | *HR* (95% *CI*) | *P* value |
| Age (years) | 1.013(0.987-1.040) | 0.335 |  | - | - |
| BMI (kg/m^2^) | 1.014(0.933-1.102) | 0.742 |  | - | - |
| ECOG score (0/1 vs. 2) | 0.920(0.576-1.467) | 0.725 |  | - | - |
| ISUP grading group |  |  |  |  |  |
| 1 | Ref | - |  | Ref | - |
| 2 | 2.140(0.603-7.598) | 0.239 |  | - | 0.590 |
| 3 | 2.545(0.752-8.612) | 0.133 |  | - | 0.682 |
| 4 | 4.654(1.423-15.226) | 0.011 |  | - | 0.391 |
| 5 | 4.691(1.343-16.389) | 0.015 |  | - | 0.606 |
| Clinical T stage |  |  |  |  |  |
| 2 | Ref | - |  | - | - |
| 3 | 0.728(0.378-1.401) | 0.341 |  |  |  |
| 4 | 1.139(0.606-2.139) | 0.687 |  |  |  |
| PSA at AA start (ng/ml) | 0.998(0.984-1.012) | 0.772 |  | - | - |
| PSA nadir after AA (ng/ml) | 1.043(1.102-1.065) | ＜0.001 |  | 1.023(1.002-1.046) | 0.035 |
| ADT duration  (<12 vs. ≥12 months) | 0.235(0.114-0.384) | ＜0.001 |  | 0.392(0.221-0.695) | 0.001 |
| Metastatic sites  (bone only vs. viscera) | 1.642(1.057-2.552) | 0.027 |  | - | 0.519 |
| SMI (low vs. high) | 0.155(0.093-0.258) | ＜0.001 |  | 0.256(0.143-0.459) | <0.001 |
| PPFA/PA (low vs. high) | 2.722(1.731-4.280) | ＜0.001 |  | 1.663(1.025-2.697) | 0.039 |

rPFS: radiological progression-free survival; mCRPC: metastatic castration-resistant prostate cancer; AA: abiraterone acetate; HR: hazard ratio; CI: confidence interval; BMI: body mass index; ECOG: Eastern Cooperative Oncology Group performance status score; ISUP: International Society of Urological Pathology; PSA: prostate-specific antigen; ADT: androgen deprivation therapy; SMI: skeletal muscle index; PPFA/PA: periprostatic fat area/prostate area.
