# Supplementary material for: Body composition parameters were associated with response to abiraterone acetate and prognosis in patients with metastatic castration‐resistant prostate cancer
Source: Cancer Med. 2023 Feb 7;12(7):8251–66. doi: 10.1002/cam4.5640 (PMC10134370; doi:10.1002/cam4.5640)
Supplement: Supplementary file 5 — Table S5 [file CAM4-12-8251-s002.docx]

| Table S5. Univariate and multivariate Cox regression analyses exploring prognostic factors for FFS in mCRPC patients receiving AA treatment | | | | | |
| --- | --- | --- | --- | --- | --- |
| Variables | FFS | | | | |
|  | Univariate | |  | multivariate | |
|  | *HR* (95% *CI*) | *P* value |  | *HR* (95% *CI*) | *P* value |
| Age (years) | 1.006(0.984-1.029) | 0.584 |  | - | - |
| BMI (kg/m^2^) | 0.980(0.913-1.052) | 0.581 |  | - | - |
| ECOG score (0/1 vs. 2) | 0.873(0.584-1.303) | 0.505 |  | - | - |
| ISUP grading group |  |  |  |  |  |
| 1 | Ref | - |  | Ref | - |
| 2 | 1.321(0.581-3.006) | 0.507 |  | - | 0.641 |
| 3 | 1.100(0.490-2.469) | 0.818 |  | - | 0.712 |
| 4 | 1.840(0.857-3.954) | 0.118 |  | - | 0.546 |
| 5 | 2.395(1.036-5.540) | 0.041 |  | - | 0.652 |
| Clinical T stage |  |  |  |  |  |
| 2 | Ref | - |  | - | - |
| 3 | 0.745(0.422-1.314) | 0.309 |  |  |  |
| 4 | 1.148(0.661-1.992) | 0.624 |  |  |  |
| PSA at AA start (ng/ml) | 1.000(0.988-1.013) | 0.946 |  | - | - |
| PSA nadir after AA (ng/ml) | 1.044(1.023-1.065) | ＜0.001 |  | 1.023(1.001-1.045) | 0.037 |
| ADT duration  (<12 vs. ≥ 12 months) | 0.333(0.223-0.498) | ＜0.001 |  | 0.503(0.317-0.797) | 0.003 |
| Metastatic sites  (bone only vs. viscera) | 1.531(1.045-2.242) | 0.029 |  | - | - |
| SMI (low vs. high) | 0.255(0.169-0.385) | ＜0.001 |  | 0.404(0.252-0.648) | ＜0.001 |
| PPFA/PA (low vs. high) | 2.432(1.616-3.659) | ＜0.001 |  | 1.616(1.043-2.502) | 0.031 |

FFS: failure-free survival; mCRPC: metastatic castration-resistant prostate cancer; AA: abiraterone acetate; HR: hazard ratio; CI: confidence interval; BMI: body mass index; ECOG: Eastern Cooperative Oncology Group performance status score; ISUP: International Society of Urological Pathology; PSA: prostate-specific antigen; ADT: androgen deprivation therapy; SMI: skeletal muscle index; PPFA/PA: periprostatic fat area/prostate area.
